# NMR Techniques in Metabolomic Studies: A Quick Overview on Examples of Utilization

**DOI:** 10.1007/s00723-016-0846-9

**Published:** 2016-11-02

**Authors:** Joanna Kruk, Marek Doskocz, Elżbieta Jodłowska, Anna Zacharzewska, Joanna Łakomiec, Kornelia Czaja, Jacek Kujawski

**Affiliations:** 1 Department of Organic Chemistry, Faculty of Pharmacy, Poznan University of Medical Sciences, Grunwaldzka 6 Str., 60-780 Poznan, Poland; 2 RootInnovation Sp. z o.o., Jana Matejki 11 Str., 50-333 Wrocław, Poland; 3 Foundation for Development of Science and Business on Medical and Exact Sciences Area, Legnicka 65 Str., 54-206 Wrocław, Poland

## Abstract

Metabolomics is a rapidly developing branch of science that concentrates on identifying biologically active molecules with potential biomarker properties. To define the best biomarkers for diseases, metabolomics uses both models (in vitro, animals) and human, as well as, various techniques such as mass spectroscopy, gas chromatography, liquid chromatography, infrared and UV–VIS spectroscopy and nuclear magnetic resonance. The last one takes advantage of the magnetic properties of certain nuclei, such as ^1^H, ^13^C, ^31^P, ^19^F, especially their ability to absorb and emit energy, what is crucial for analyzing samples. Among many spectroscopic NMR techniques not only one-dimensional (1D) techniques are known, but for many years two-dimensional (2D, for example, COSY, DOSY, JRES, HETCORE, HMQS), three-dimensional (3D, DART-MS, HRMAS, HSQC, HMBC) and solid-state NMR have been used. In this paper, authors taking apart fundamental division of nuclear magnetic resonance techniques intend to shown their wide application in metabolomic studies, especially in identifying biomarkers.

## Introduction

**Fig. 1:**
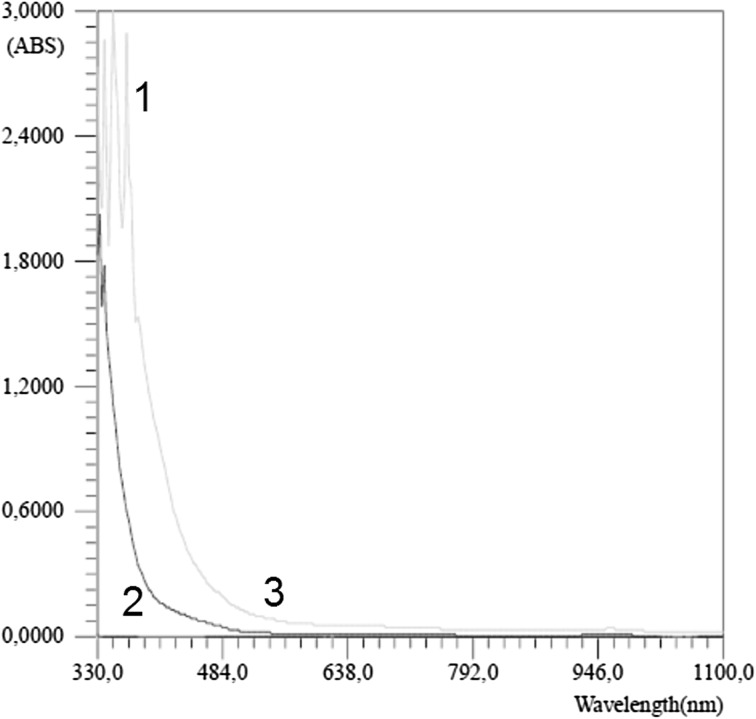
UV–Vis spectrum of human urine. *1* Water, *2* 6-year-old kids, *3* adult. Own researches

Metabolomics is a rapidly growing branch of science and medicine that aims at identifying new biomarkers of a variety of human diseases and disorders, also investigating them on animal models. It focuses primarily on diseases for which currently no definite biomarker is known. The techniques most commonly utilized in metabolomics are: mass spectrometry (MS) as an analytical technique, together with gas or liquid chromatography (GC or LC) as additive methods, or nuclear magnetic resonance (NMR) spectroscopy. Other methods are: UV–VIS (Fig. 1), IR, RAMAN and SEM [1]. Whereas mass spectroscopy measures the ratio of mass to charge of ionized particles, nuclear magnetic spectroscopy takes advantage of the magnetic properties of certain nuclei, such as ^1^H, ^13^C, ^31^P and others [2–4]. This spectroscopy consists in arousing nuclear spins being located in a magnetic external field through rapid changes of the magnetic field, and then recording of the electromagnetic radiation occurring as a result of occurrences of the relaxation. When a given frequency of the electromagnetic wave is used, only the nuclei with such resonance frequency absorb it. The immediate surroundings of the nucleus affect its resonance frequency (frequency for which pulses have the largest amplitude), thus making it possible to distinguish nuclei, which are surrounded by different atoms in a given compound.

We intend to show wide application of nuclear magnetic resonance techniques in metabolic studies based on crucial division on one-dimensional, two-dimensional, three-dimensional and solid-state NMR (ssNMR) techniques. We also briefly present examples of ^19^F NMR spectroscopy techniques from the discussed standpoint; however, it is noteworthy that due to number of literature data regarding this method, this topic will be a goal of further review. To facilitate broadening knowledge about spectroscopy and metabolomics, fundamental abbreviations are presented (Table 1).

**Table 1:** List of abbreviations used in current publication

Abbreviation	Technique
NOEPR	1D Nuclear Overhauser Effect spectroscopy, Pulse train with Presaturation during Relaxation and mixing time
WET	Water suppression Enhanced through T1 effect
ES	Excitation sculpting
ESCW	CW on-resonance saturation pulse
ESWGL	Adiabatic frequency modulation “wiggly” pulse during the relaxation delay
MISSISSIPPI	Multiple Intense Solvent Suppression Intended for Sensitive Spectroscopic Investigation of Protonated Proteins, Instantly
CPMG	Carr–Purcell–Meiboom–Gill
HRMAS	High-Resolution Magic-Angle Spinning
PASS	Phase-Adjusted Spinning Sidebands
PHORMAT	PHase-cORrected Magic-Angle Turning
MACS	Magic-Angle Coil Spinning
ERETIC	Electronic REference To access In vivo Concentrations
CP	Cross-Polarization
DEPT	Distortionless Enhancement by Polarization Transfer
q-MAS PGSE	Magic-Angle Spinning of the Q-vector in Pulsed-Gradient Spin-Echo

### One-Dimensional Techniques

The most popular NMR technique is ^1^H NMR based on ^1^H nucleus because of their abundance in nature (over 99.98%) [5], low relaxation time and an appreciable nuclear spin.

Because this method has been commonly used in metabolomics of different biofluids for years, there are numerous emerging techniques involving NMR that are particularly useful in this area [2, 3]. There are many reviews concerning the latest advances in this branch of science [4, 6], but to the authors’ knowledge, there is none regarding the types of NMR techniques and approaches used for metabolomic studies [7, 8], and the authors aim at filling this gap. Moreover, we want to show why these techniques were introduced, what they help to achieve and which challenges they help to overcome. Apart from ^1^H NMR, the techniques employing other nuclei will also be included, such as ^13^C [9], ^31^P [10], ^43^Ca [11].

**Scheme 1:**
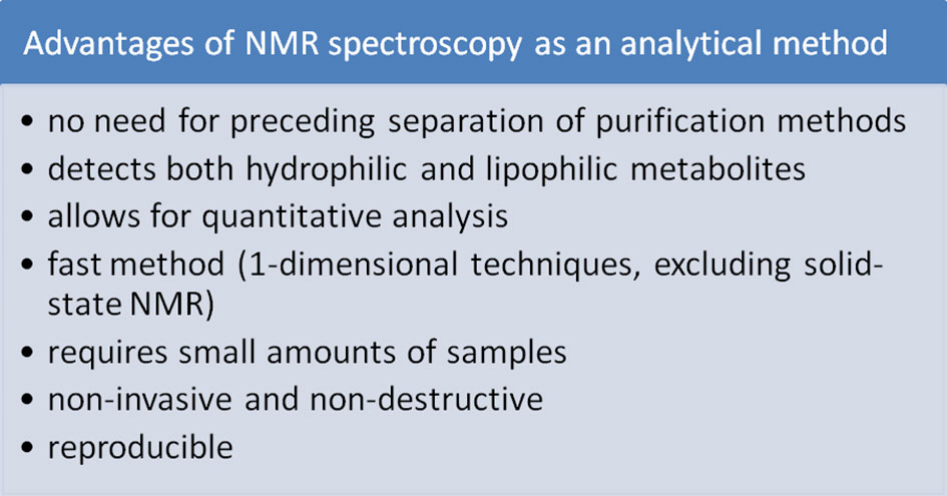
Modified according to Refs. [2–4, 6–13, 20–22, 25, 73–76]

NMR spectroscopy has numerous advantages listed in Scheme 1. However, it is capable of detecting the compounds given above micromolar concentrations. To perform the analysis, liquid-state NMR requires an addition of an external standard; for example, tetramethylsilane (TMS) or 4,4-dimethyl-4-silapentane-1-sulfonate (DSS) in case of using water as solvent or octadecanoic acid and methyl octadecanoate for fatty acids and theirs derivatives [12–14]. To perform the quantitative analysis, for example, method utilizing the area per proton (determined by integration) [14], LCModel [15] or OPLSA-DA strategy [16] is used. Quantitative analysis is somewhat more challenging in ssNMR, nevertheless, it is also viable. The examples of using HRMAS technique in intact brain tissue [17] or biopsies from patients suffering from breast cancer [18] analyses show the plenty of other application. Moreover, the detection of metabolites is relatively fast in one-dimensional NMR (however, this does not adhere to most ssNMR techniques). Furthermore, this approach requires only small amounts of samples, which is crucial in metabolomics, insofar as usually only small amounts of biofluids or tissues are available. In addition, it is not invasive and destructive, which means that after analysis tissues could be used for further analyses (biofluids as well, but only if the external standard in liquid-state NMR and pH adjustment would not impact the results). Therefore, the ^1^H NMR spectroscopy is used in metabolomics primarily inasmuch as enable quantitative, reproducible and usually relatively analysis [15, 16, 19].

One-dimensional NMR spectroscopy is the one that is most commonly used, but certain problems can be encountered during such analysis as ^1^H NMR spectra contain many overlapping peaks. One compound that causes problems is glucose. In case of this sugar, strong peaks between 3.2 and 4.0 ppm overshadow other signals in this part of the serum or urine spectrum. However, these difficulties can be overcome for instance by an “Add to Subtract” background subtraction method [20]. According to the obtained results, the glucose signals can be decreased by 98%, which allows to obtain the otherwise invisible data. The procedure involves acquiring a second spectrum after adding a small amount of concentrated glucose solution to the sample. Then the differences between the NMR spectra with and without added glucose can be computed. Other methods that allow to remove glucose signals without influencing other signals are background subtraction and separation techniques prior to the NMR analysis [20–23].

Not only glucose or proteins [23, 24] signals can overshadow other peaks—the most abundant compound in the NMR metabolomics is water, the main component of biofluids and tissues. Therefore, water suppression is crucial to obtain interpretable spectra and there are numerous water suppression and water presaturation techniques. In a comparison of NOEPR (1D NOESY, Nuclear Overhauser Effect Spectroscopy, Pulse train with Presaturation during Relaxation and mixing time), WET (Water suppression Enhanced through T1 effect) and excitation sculpting (accompanied by either a CW on-resonance saturation pulse, ESCW, or an adiabatic frequency modulation ‘‘wiggly’’ pulse during the relaxation delay, ESWGL), it was concluded that ESCW, ESWGL, NOEPR and ES methods obtain great water suppression with high reproducibility. WET yielded distinctly worse results [21]. It is worth mentioning that NOESY technique is the most popular and enables optimization of process. Other solvent suppression techniques are being developed, such as Multiple Intense Solvent Suppression Intended for Sensitive Spectroscopic Investigation of Protonated Proteins, Instantly (MISSISSIPPI), which consists of gradient and saturation pulses [22]. Currently, due to the variability in the quality of water suppression, the water peaks are present in the spectra and, therefore, the region of water resonance is not included in the metabolomic analysis (approximately 4.50–5.00 ppm). The emerging water suppression techniques might change it and make it possible to retrieve data also from this part of the spectrum [22].

**Fig. 2:**
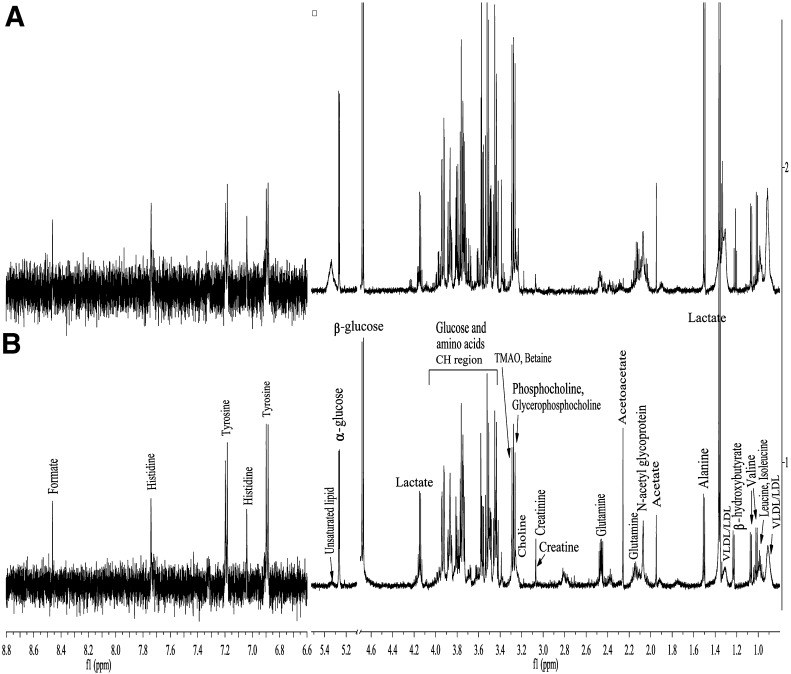
Example of one-dimensional Carr– Purcell–Meiboom–Gill (1D – CPMG) 1H NMR spectra of serum spectra from: **a** healthy patient, **b** patient suffering from esophageal cancer showing key metabolites. Modified according to [25]

1D spectra are also acquired with the Carr–Purcell–Meiboom–Gill (CPMG) pulse sequence to decrease broad signals from proteins and lipoproteins because of their relatively long transverse relaxation times [23, 25]. Thus, the signals from compounds with low molecular weight are not overshadowed by signals of macromolecules. An example of mentioned spectrum is given in Fig. 2.

### Two-Dimensional (2D) Techniques

The problems that arise from signal overlap can be partly overcome by employing two-dimensional NMR spectroscopy. It allows to determine the peak intensity and its properties, such as the spin multiplicity and coupling constant, even if in 1D spectra it is overshadowed by a stronger signal. Table 2 outlines a concise comparison of 1D and 2D NMR techniques. There are a number of 2D NMR methods that are particularly commonly used in metabolomics to determine the presence and concentrations of otherwise undetectable compounds or confirm peak assignment by spin connectivities [26]. In Table 3 are depicted the most commonly used 2D NMR techniques with their application in metabolomics. So far, there is not known a fast and automatic methodology of signal’ deconvolution and times of measurements are not acceptable.

**Table 2:** Comparison of parameters of 1D and 2D NMR techniques

Parameter	1D	2D
Degree of overlap for quantitative analysis	Higher	Lower
Sensitivity to pulse imperfections	Lower (lower signal to noise ratio)	Higher
Number of factors influencing peak	Lower	Higher (e.g., J-coupling or relaxation time)
Calibration procedure	One for the whole experiment	Individual for each compound (for quantitative analysis—peaks ratio does not reflect the concentration ratio directly)
Experiment duration	Shorter	Longer
Efficacy (particularly for small molecules)	Lower	Higher

Modified according to Refs. [26, 55]

**Table 3:** The most commonly used 2D NMR techniques with their application in metabolomics

Method	How it works	What can we do with it?	Subtypes, other types of this method
COSY (homonuclear correlation spectroscopy)	The first 2D NMR method used for the analysis of extracts Identifies spins that are coupled to one another	Facilitates identification of: GABA (the pattern of its 3 methylene protons), glycerophosphocholine, phosphoethanolamine and myoinositol (spin connectivities) imidodipeptides (those including proline or hydroxyproline) [56]	Double Quantum Filtered shift-COrrelated SpectroscopY (DQF-COSY)-enhanced spectral resolution, better determination of the coupling constant, suppression of large singlets [21] COCONOSY (NOE and shift-correlated spectroscopy): confirmation of malate peak assignment [26]
HSQC (Heteronuclear Single Quantum Coherence)	Uses magnetization transfer between nuclei, usually between hydrogen and carbon atoms (^1^H–^13^C) [57] Not a quantitative technique T1 noise artifacts present, but can be corrected with algorithms [58]	Confirmed identification of: Numerous aminoacids (isoleucine, leucine, valine, threonine, lysine, glutamate, proline, glutamine, asparagine, phenylalanine) Lactate and glucose Ethanolamine and glycerol Glycogen Lipids [59]	Adiabatic pulsing—generates quantitative data gHSQC [60] Offset-compensated, CPMG-adjusted HSQC (Q-OCCAHSQC) [61] Q-HSQC QQ-HSQC (shorter total acquisition time) Q-CAHSQC HILIC (2D Hydrophilic Interaction Chromatography), can replace LC-NMR to identify certain metabolites, e.g., in urine [62] HSQC0 (time-zero 2D spectrum) signal intensities are proportional to compounds’ concentrations, which facilitates their identification [63]
HMQC (Heteronuclear Multiple-Quantum Correlation)	Broader peak because of homonuclear proton J-coupling (worse resolution) More difficult metabolite identification [54]	Assigning peaks of 1- and 3-methylhistidine	
NOESY (Nuclear Overhauser Effect Spectroscopy)	Has additional peaks that are not informatory (they can be eliminated by reversing the phase) The resonances of nuclei which are closely located in space are coupled to each other (not those which are connected by covalent bonds)	Confirmation of peak assignment in 1D spectra [64] For certain solute concentrations better water suppression than the one achieved by standard water presaturation techniques [65]	
DQF-COSY (Double Quantum Filtered shift-COrrelated SpectroscopY)	Uses the phenomenon of double quantum coherence between scalar-coupled protons	Improved spectral resolution Identification of sugar protons and coupled methylene protons Suppression of big singlet signals, and thanks to it, reduction of spectral artifacts from T1 noise Facilitates determination of the spin multiplicity and the coupling constant [26]	
HMBC (Heteronuclear Multiple Bond Coherence)		Less frequently used than HSQC Suppression of the HSQC-like one-bond interaction is not always achieved, which might result in artifacts [66]	
INADEQUATE (Incredible Natural Abundance Double Quantum Transfer Experiment)		Reduced overlap [55] Identification of taurine, myoinositol, serine thanks to diagonal transparency (not possible in TOCSY/COSY) [67] Less effective resonance discrimination than HSQC [68]	
TOCSY (TOtal Correlation SpectroscopyY	Cross peaks are formed for both directly and indirectly coupled nuclei	A clearer spectra than in 1D NOESY spectra with reduced spectral overlap [69] Facilitation of determination of nucleotides (e.g., uridine nucleotides) thanks to reduction of spectral overlap [70]	
STOCSY (Statistical TOCSY)		Generates a pseudo-2D spectrum, which shows the relationships between the intensities of different peaks [71]	SHY-statistical heterospectroscopy for the coanalysis of multispectroscopic data of a number of samples [71]
2D JRES NMR (two-dimensional J-resolved NMR spectroscopy)		It is a reliable and fast technique Measures isotopic patterns of compounds labeled with ^13^C carbon Suitable for reproducible isotopic profiling (e.g., of isotopomers of alanine) [72]	p-JRES NMR (projections of 2D J-resolved NMR have more uniform baseline and the peaks are less crowded than in 1D spectra) [72]

Modified according to Refs. [26, 54, 56–72]

**Fig. 3:**
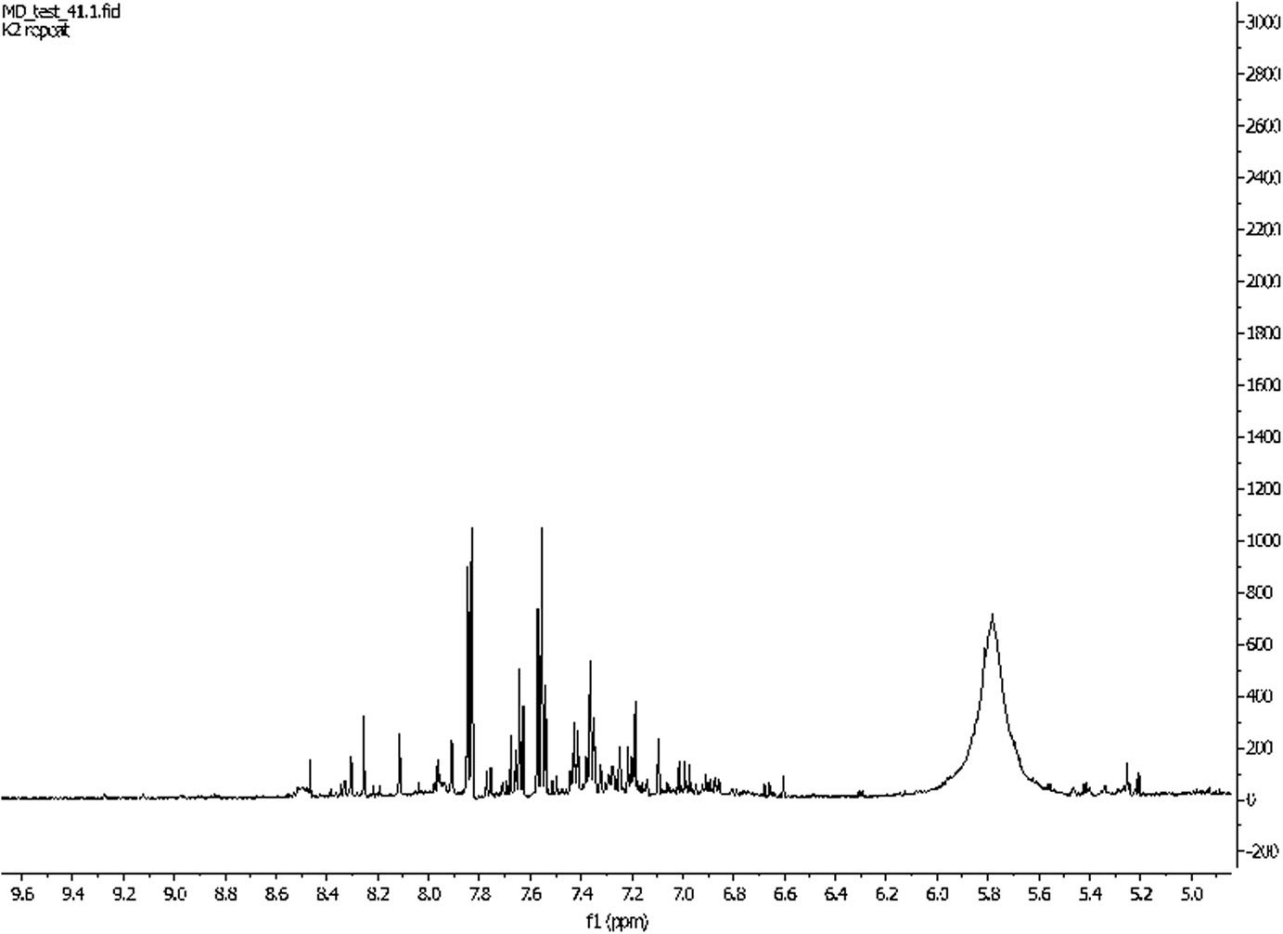
1H NMR spectrum of human urine. Patient treated by cisplatin. Own researches

**Fig. 4:**
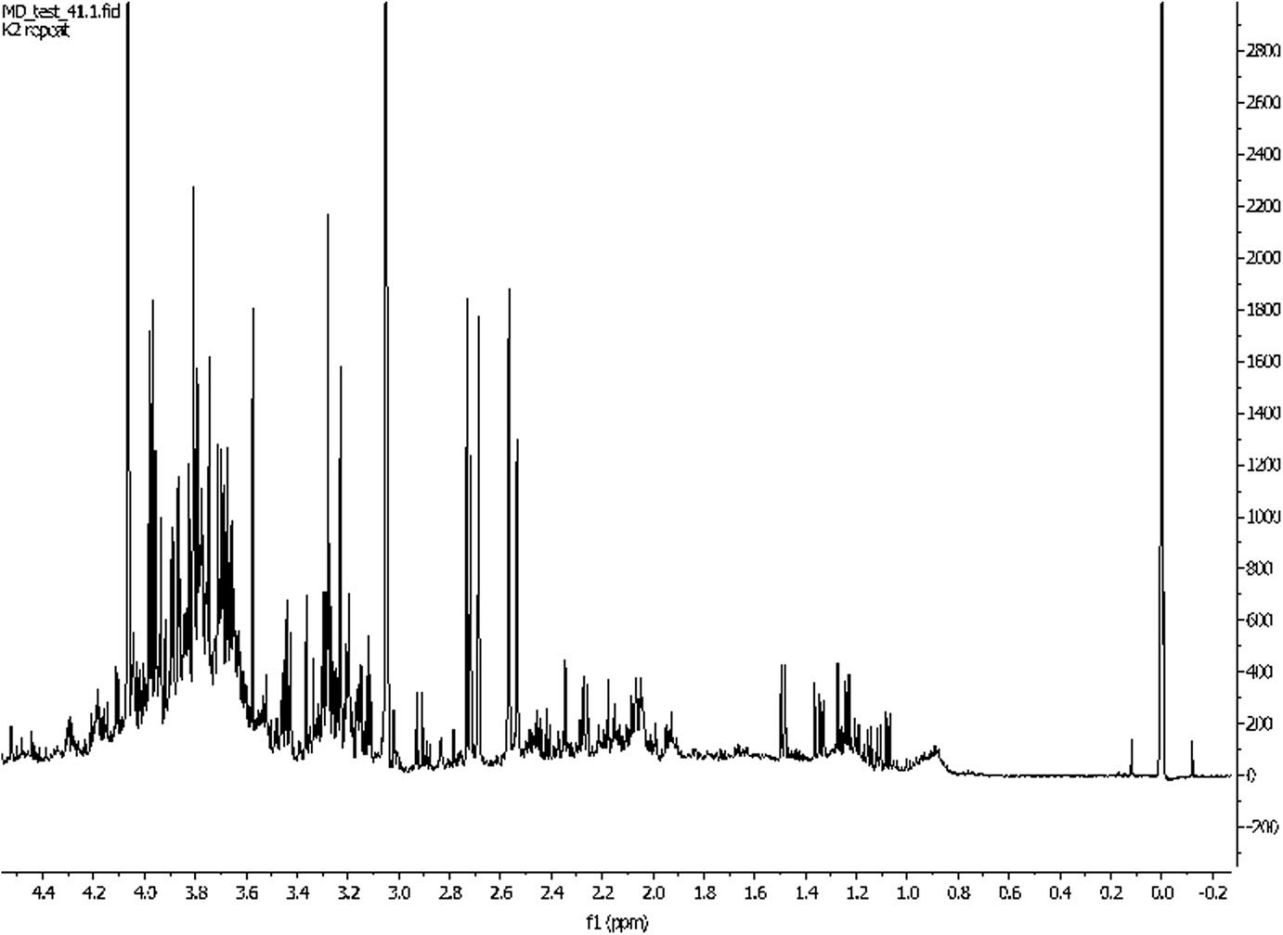
1H NMR spectrum of human urine extension. Patient treated by cisplatin. Own researches

**Fig. 5:**
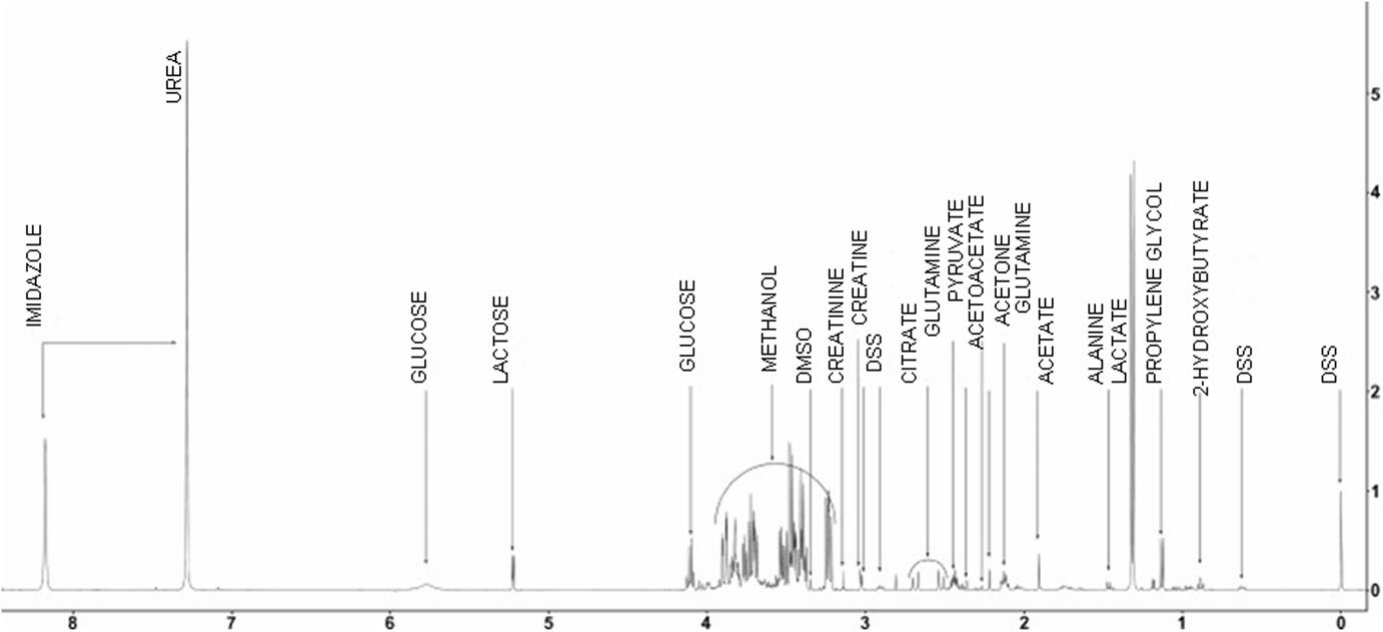
1H NMR spectrum of human CSF. Modified according to Ref. [28]

It is noteworthy that the difficulties encountered during analysis depend on properties of biofluid tested. The most widely used biofluids in metabolomics are serum/plasma and urine (Figs. 3, 4). They are relatively easy to obtain and their collection is relatively non-invasive. Additionally, the cerebrospinal fluid (CSF) is also examined [27], but on a smaller scale because of the invasiveness of the lumbar puncture. Herein, the fact that NMR analysis requires only small amounts of samples is particularly important, inasmuch as bigger amounts of CSF would be extremely difficult to obtain without side effects for the patient. On the other hand, this small amount of sample is sufficient for detecting crucial substances (Fig. 5) [28].

Appropriate identification of compounds and determination of their concentrations is of utmost significance. Furthermore, also a dataset of ‘control’ NMR biofluid results needs to be gathered so as to have a reference group that can be compared to those with a particular disease. It is difficult, inasmuch as compounds of low molecular weight can easily be disturbed by a number of factors, not only a given disease or treatment. Therefore, it is crucial to establish the NMR metabolome of human serum, urine and the cerebrospinal fluid, which is primarily done using 1D NMR techniques [28–30].

**Fig. 6:**
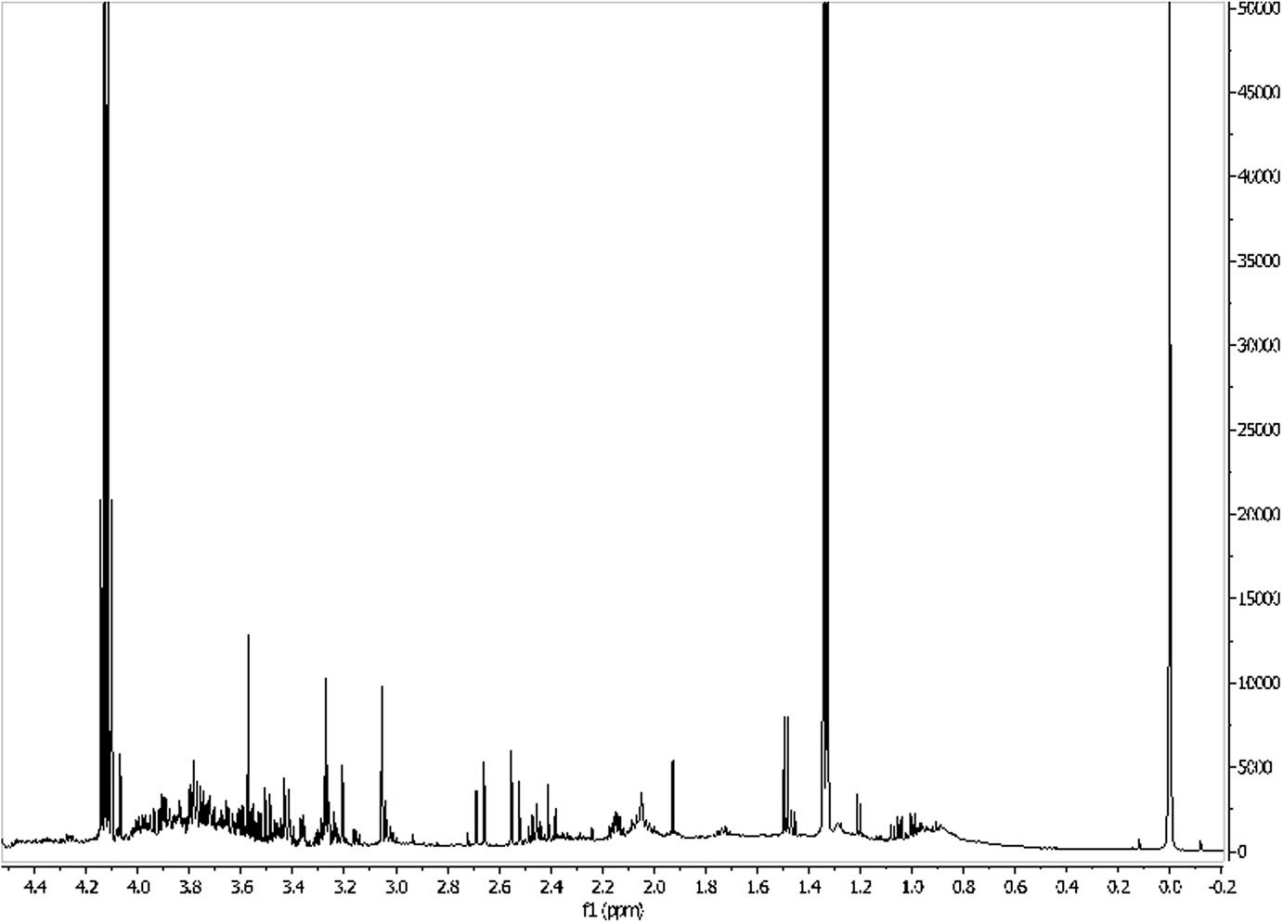
1H NMR spectrum of the human waters (0–5 ppm). Own researches

**Fig. 7:**
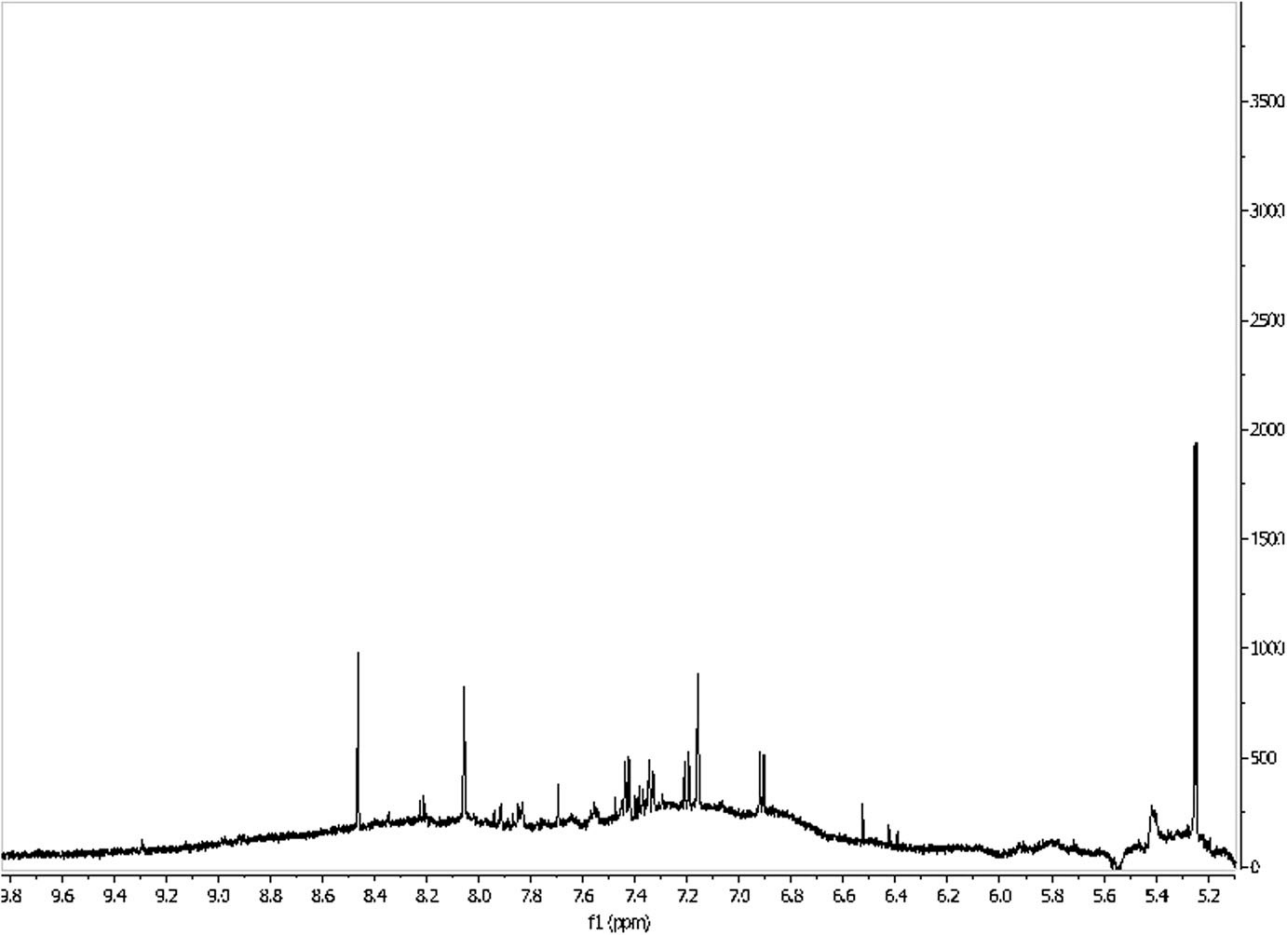
1H NMR spectrum of the human waters (5–10 ppm). Own researches

By NMR techniques it is possible to identify the most compounds in the metabolome of, for example, urine, blond, saliva, sweat, tears, the waters (Figs. 6, 7) and CSF. Generally, the number of identified compounds is higher in comparison to other techniques, such as gas chromatography–mass spectrometry (GC–MS) and high-performance liquid chromatography (HPLC) [31]. For instance, both for the metabolome of CSF and urine NMR detected the most compounds, with the runner-up being GC–MS. NMR is also useful in investigating the serum metabolome [29]. Therefore, it seems to be the most appropriate tool for general metabolomic analyses, despite its sensitivity only to compounds above micromolar concentrations [28]. An additional advantage of using NMR for ultrafiltered serum metabolomics is the relative simplicity of the spectra and, therefore, lack of difficulties with peak assignment [29].

Urine metabolome was also investigated on fractions from solid-phase extraction (SPE), with the NMR spectra obtained using a 1D-NOESY pulse sequence with presaturation [30]. There, SPE allowed to obtain five different fractions that contained compounds with different polarity. Whereas the first fraction contains the most polar metabolites, the fifth one contains the least polar ones. As urine primarily consists of polar compounds, the most metabolites were identified in the first fraction and the fewest in the last three ones (almost seven times fewer than in the first fraction).

Additional information on the metabolome of urine can be obtained by means of proton decoupled 13C{1H} NMR spectroscopy by aiding peak assignment, difficult in 1D NMR spectra because of peak overlap [32]. A further advantage of this technique is lack of water signals. Therefore, no water suppression is necessary in this approach. On the other hand some disadvantages, such as low sensitivity and long time of experiment, can be pointed out.

## Solid-State NMR (ssNMR)

**Fig. 8:**
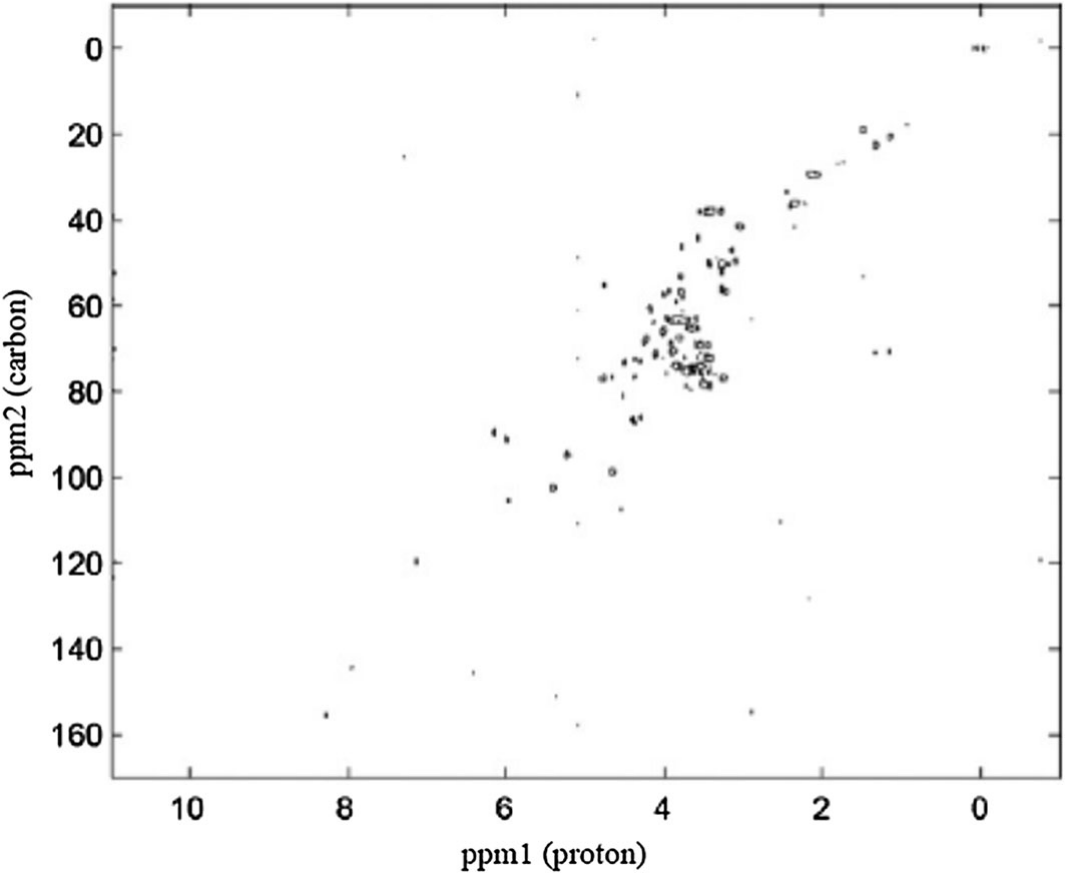
Example of 2D 1H, 13C HSQC NMR spectrum of biological sample—an extract of fish eggs. Modified according to [35]

Apart from body fluids and tissue extracts (Fig. 8), metabolomics also allows to examine tissue samples, but separate methods have to be used then, such as ssNMR. The advantages of such spectra is the fact that they require little preparation and they retrieve results comparable to those obtained in NMR of considerably more time-consuming tissue extracts. Furthermore, ssNMR is a non-destructive technique and require small tissue samples. Being non-destructive is particularly important in solid-state NMR as it means that after NMR analysis the tissue can be sent for histopathology examination. Obviously, such spectra comprise numerous different peaks and, therefore, 2D NMR techniques also have to be used. However, in solid-state spectra due to dipolar coupling and chemical shift anisotropies, the obtained signals are broadened and, therefore, the signal overlap is increased and they are more difficult to identify. At first, these difficulties were overcome by isotope labeling (particularly in collagen research); nevertheless, it was a costly and time-consuming method. This is why currently in solid-state experiments, high-resolution magic-angle spinning (HRMAS) spectroscopy is used. It decreases line broadening by rotating the sample at the angle of 54.74° (the ‘magic angle’) with respect to the external magnetic field, thus eliminating the anisotropic interactions. It is necessary to use high spinning rates because they have to be higher than or similar to the interaction strength: and especially the dipolar couplings between two hydrogen atoms are quite strong. When lower spinning rates are used, a series of narrow peaks is obtained, separated by equal intervals (equal to multiples of the applied frequency of rotations) [33].

HRMAS techniques allow to achieve better resolution; however, the factors influencing line broadening are highly dependent on the material structure and enable, for example, investigation of bone mineralization [33]. This is why there are few methods that make it possible to conduct recoupling of the anisotropic Hamiltonians and to separate the anisotropic and isotropic interactions, thanks to which more comprehensive data can be obtained that inform us about the material structure [33].

Nevertheless, the spinning in MAS may cause cell and tissue destruction both by rotations and an increase of temperature, and thus impact the measurements. This is why in cases of particularly vulnerable specimens, slow MAS can be used. Obviously, then an additional method must be used to suppress the spinning sidebands, which is normally achieved by fast MAS. Such methods are phase-adjusted spinning sidebands (PASS) and phase-corrected magic-angle turning (PHORMAT). They can be used with magic-angle coil spinning (MACS) to examine small samples with high sensitivity [75].

A challenge, not encountered in NMR of solutions, presents itself in solid-state NMR—quantification of metabolites. In solid-state NMR, it is very difficult or even impossible to add an exact amount of a reference compound. Nonetheless, a method was developed that makes it possible to overcome this obstacle, namely ERETIC (Electronic REference To access In vivo Concentrations). Since its development, it has become widespread in HRMAS NMR as a method that allows to quantify the detected metabolites, e.g., in brain tissue examinations or breast cancer biopsies [17, 18].

**Fig. 9:**
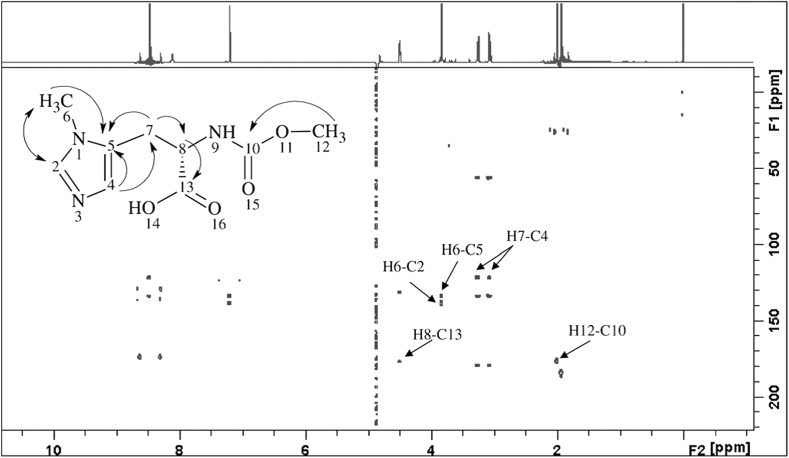
Example of proton–carbon cross-correlation shown in the HMBC two-dimensional NMR spectrum. Modified according to [54]

Apart from tissue samples, such as prostate, cancer biopsies and other organs, also bone and cartilage are researched using HRMAS spectroscopy. Particularly for bone, because of its high phosphate content, cross-polarization HRMAS spectroscopy is used to obtain ^31^P spectra of better resolution. It uses magnetization transfer from abundant nuclei (such as ^1^H) to those less abundant (for example ^31^P) via dipolar couplings to enhance the signals from the less numerous ones. However, as was mentioned, fast spinning rate decreases heteronuclear dipolar couplings (such as those between ^1^H and ^31^P atoms)—which impairs CP. This way, signals from atoms other than hydrogen that do not have strong couplings to hydrogen nuclei can be eliminated [35]. Because of these relationships, the signal intensity is dependent on the time during which the polarization transfer takes place. This is why in such experiments signal intensity must be compared over a variety of contact times, and not only for its fixed value. Such investigations were carried out considering the phosphorus components and their structure in bone (the graph of intensity vs. contact time is strongly dependent on the material structure) [36–39] and bone implants [33] (Fig. 9). Cross-polarization can be improved using multiple cross-polarization pulse sequences, which considerably shorten the acquisition time [76].

The time required to obtain the spectra in ssNMR can be decreased, e.g., using paramagnetic doping. Then an addition of a solution of copper (II) ions to powdered tissues containing collagen prior to ^13^C CP MAS resulted in a shortening of the hydrogen spin–lattice relaxation time and thus shortened the time needed to acquire the spectrum (because it is highly dependent on this relaxation time). It did not impair the spectral resolution [41].

Apart from CP, also distortionless enhancement by polarization transfer (DEPT) can be applied to improve MAS NMR sensitivity. In liquid-state NMR, it is used to distinguish signals of methyl, methylene and methine groups in ^13^C NMR spectra. When compared to other techniques, this solid-state method only enhances the signals of compounds of low molecular weight, whereas lipid molecule signals are primarily improved when using the CP technique because of their low mobility in ^13^C MAS [42].

With HRMAS it is also possible to measure the diffusion coefficient and relaxation times (so-called *T*_1_ and *T*_2_). Due to these parameters, signals of low molecular weight compounds can be eliminated because of their longer relaxation time *T*_1_. Then, lipid signals are enhanced and easier to interpret without using time-consuming 2D NMR methods. Therefore, diffusion-weighted pulse sequences are a different approach which makes it possible to identify mobile lipid resonances without the overlap with peaks of low molecular weight metabolites [43]. A conjoint measurement of apparent diffusion coefficients and *T*_2_ can also be measured when one aims at enhancing the peaks of low molecular weight compounds. Then for this purpose an additional gradient spin**-**echo sequence might be used [44].

New, emerging techniques include comprehensive-multiphase NMR (CMP-NMR), which allows to investigate structures present in each phase and the interaction between the phases. So far, it has been investigated with soil samples, but it might prove to be a great tool in tissue examination, especially to study the complex interactions between the different tissue components (such as the blood cells and plasma) [45]. Another technique is q-MAS PGSE NMR (magic-angle spinning of the q-vector in pulsed-gradient spin-echo NMR), which is used to examine the pores of different materials by investigating the diffusion of water, without the contribution of anisotropic interactions (they are nullified). Furthermore, comparing q-MAS PGSE NMR and PGSE NMR results makes it possible to determine whether microscopic diffusion anisotropy is present in the examined system. In metabolomics, it might find applications in researching the structure of axons [46].

## ^19^F NMR Spectroscopy in Metabolomic Studies

In our paper, we described some ^1^H NMR spectroscopy applications; nevertheless, the progress of ^19^F spectroscopy approaches in metabolomics requires attention herein. The nuclei of ^19^F is characterised by gyromagnetic ratio almost equal to that of the proton and broader range of chemical shift. Due to connection between metabolomics and pharmaceuticals studies, we would like to indicate some achievements [47–53].

An experiments using glioma model (similarity to human glioblastoma) demonstrate that ^19^F MRI in combination with ^1^H MRI can selectively map the bio-distribution of ^19^F-BPA (boronophenylalanine). The ^19^F BPA monitoring uptake in tumors by ^19^F imaging indicates the ^19^F MRI as a tool of better understanding and investigating the pharmacokinetics of fluorinate-containing drugs. The results of experiments give information about optimal timing for neutron irradiation. In boron neutron capture therapy (BNCT), the exposure time is very important and conclusions from quoted experiment can be useful while conducting therapy [47].

^1^H, ^19^F, ^13^C and ^10^B magnetic resonance spectroscopy for another boron neutron capture therapy agent, an ^19^F-labeled, ^10^B-enriched *p*-boronophenylalanine–fructose complex (^19^F-BPA–fr) is also reported [48]. The aim of the cited study was a reaction yield optimization for the synthesis and the complexation of the BNCT agent. In addition, the feasibility of using ^19^F-MRS to perform pharmacokinetic studies of the ^19^F-BPA–fr complex was proven [49].

Moreover, application of ^19^F NMR spectroscopy in gene therapy is reported, which is limited by difficulties in assessing the success of transfection in terms of spatial extent, gene expression and longevity of expression [49]. Cui et al. prepared the molecular and ^19^F NMR characteristics of PFONPG (4-fluoro-2-nitrophenyl-β-d-galactopyranoside) in solution, blood and prostate tumor cells and showed new possibilities into developments of the gene therapy [49].

Due to the popularity of ^19^F spectroscopy in environmental studies and metabolic screening of new isolated organisms, it was used in exploring the aerobic microbial degradation [50]. By analyzing reaction of fluorophenols, the potential of ^19^F biodegradation studies was proven. Other studies explore the toxicity of a fluorine-labeled derivative of ascorbic acid (F-ASA), a major antioxidant, by measuring in vitro and in vivo accumulation of F-ASA, fluoro-dehydroascorbate or fluoro-2,3-diketogulonate and their degradation products [51]. The importance of such discoveries will be noticed when we make ourselves aware that a lot of drugs contain the fluorine atom in their structure. Many anesthetics, chemotherapeutic agents and molecules with high oxygen solubility for respiration and blood substitution can be monitored by MRS techniques, as well as by fluorine (^19^F) MRI [52]. It is worth to mention that the application of ^19^F spectroscopy techniques in the specific targeting, imaging of cellular surface epitopes, cell tracking of endogenous macrophages or injected immune cells is reported as well [52].

Compared to traditional attempt at exploring new biologically active compound, another approach is possible thanks to fluorine NMR-based assay *n*-FABS (*n*-fluorine atoms for biochemical screening), which enables screening and identification of inhibitors for a specific enzymatic target [53]. Due to easy setup, versatility and lack of interfering signals, this method might be widely applicable and should facilitate the identification and characterization of small molecule inhibitors for example of the membrane-bound serine amidase or fatty acid amide hydrolase [53].

## Conclusions

**Fig. 10:**
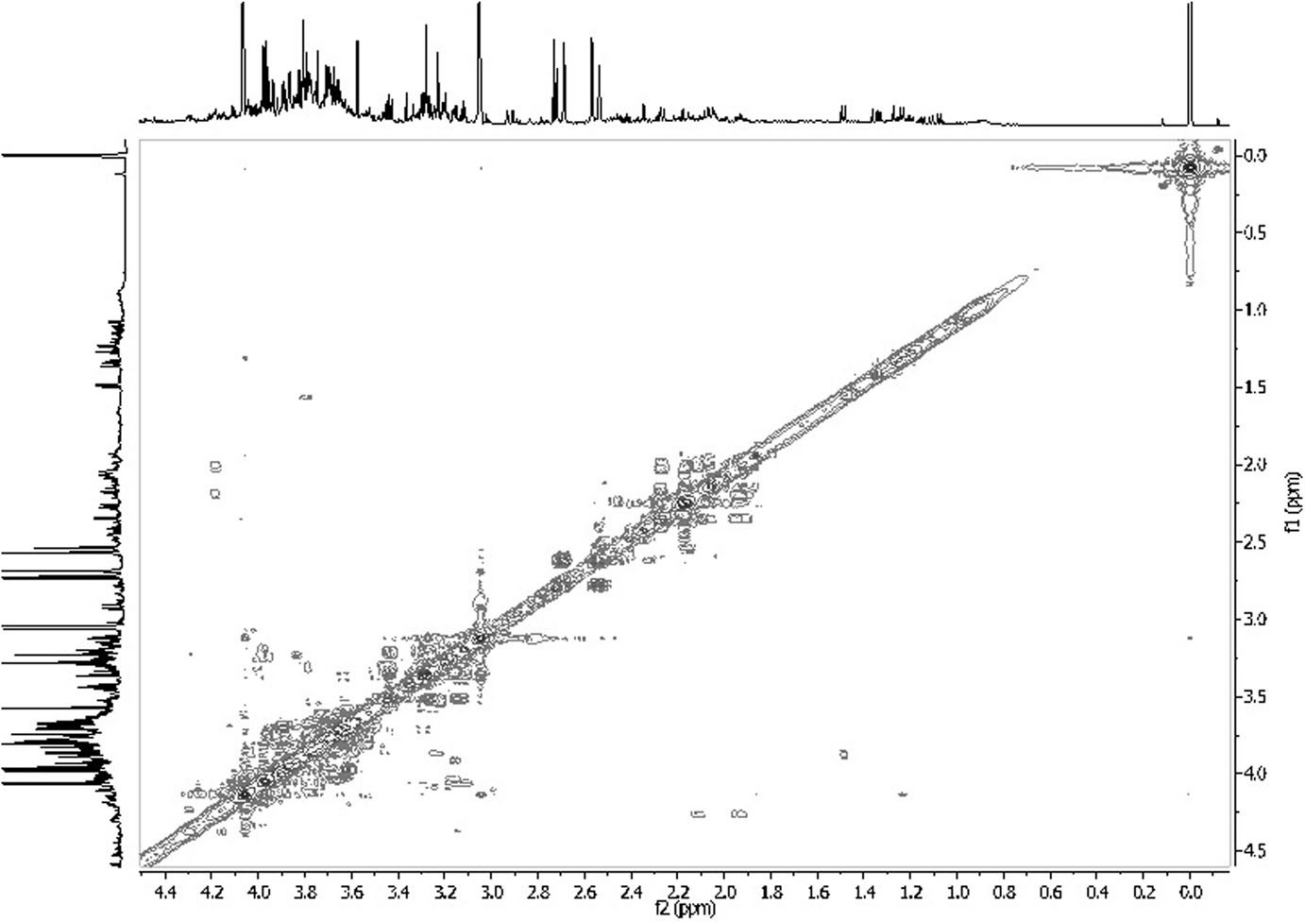
^1^H^1^H COSY spectrum of human urine. Own researches

**Fig. 11:**
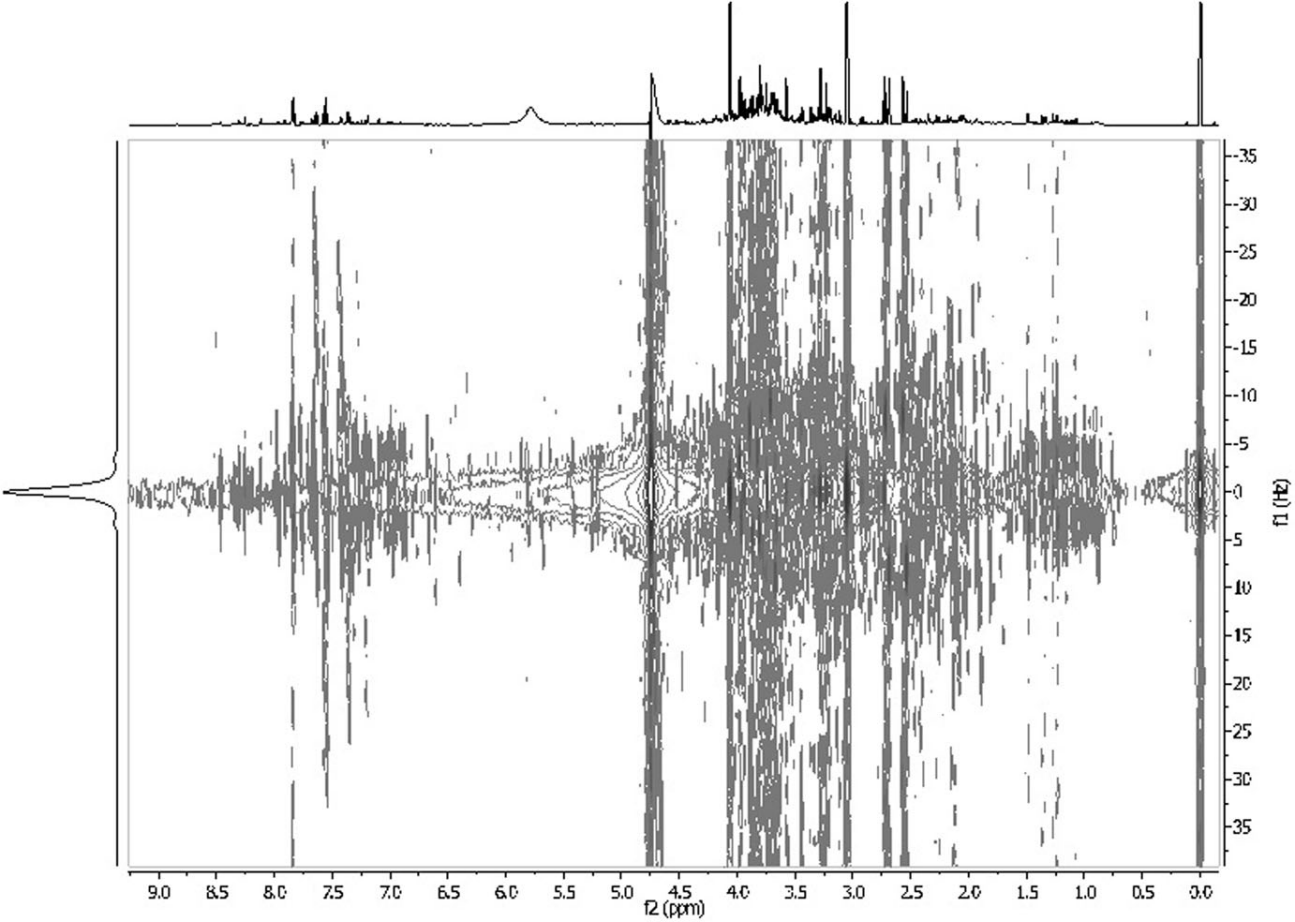
JRES spectrum of human urine. Patient treated by cisplatin. Own researches

Since years an enormous development of nuclear magnetic resonance has been observed and many explored techniques have been used in metabolomic studies. Among advantages of 1D techniques applied in metabolomics, employing nuclei such as ^13^C, ^31^P, ^43^Ca, possibility of quantitative analysis, needles of only small amounts of samples and obtaining excellent quality of spectra thanks to solvent suppression techniques are mentioned. Moreover, using 2D techniques enables analyzing various biofluids and detecting small amount of numerous substances (Figs. 10, 11). Finally, solid-state techniques are non-destructive for biological samples and generally faster than techniques mentioned above. ^19^F NMR spectroscopy application in BNCT, gene therapy or potential drug screening is also pointed out. It is believed that due to these advantages and temporary development of spectroscopic techniques, they will be still used in metabolomic studies enabling detecting new substances with potential biomarker properties.
